# COVID-19 CASE AND MORTALITY RATES IN GREEN HOUSES AND TRADITIONAL NURSING HOMES IN NEW YORK STATE

**DOI:** 10.1093/geroni/igac059.1781

**Published:** 2022-12-20

**Authors:** Yuchi Young, Ashley Shayya, Thomas O'Grady, Ya-Mei Chen

**Affiliations:** SUNY at Albany School of Public Health, Rensselaer, New York, United States; SUNY at Albany School of Public Health, Rensselaer, New York, United States; SUNY at Albany School of Public Health, Rensselaer, New York, United States; National Taiwan University, Taipei City, Taipei, Taiwan (Republic of China)

## Abstract

Introduction. Green Houses (GHs) have features that distinguish them from traditional nursing homes (NHs) including small size, home-like settings, humane model of care, and a sense of community. Literature shows these features have contributed to lower staff turnover, higher resident satisfaction, and lower COVID-19 case and mortality rates. Few studies use longitudinal data to quantify the differences between GHs and NHs by examining COVID-19 case and mortality rates. Methods. Nursing Home COVID-19 Data from CMS were used to compare case and mortality rates between GHs (n=4) and NHs (n=614) from 5/2020 to 1/2022. Case and mortality rates were calculated for GHs and NHs. Incidence rate ratio (IRR) of case and mortality rates were provided. Results. The preliminary results indicate GHs have lower COVID-19 case (3.76 vs. 6.8 per 1,000 resident weeks) and mortality rates (0.35 vs 1.21 per 1,000 resident weeks) compared to NHs. The IRR for COVID-19 cases is significantly higher in NHs compared to GHs (IRR = 1.8; 95% CI 1.55, 2.11), likewise, for mortality (IRR = 3.45; 95% CI 2.09, 5.75). Conclusions. The findings illuminate key differences in COVID-19 case and mortality rates among GHs and NHs. Factors such as GH size and their unique care model may contribute to the differences observed in COVID-19 case and mortality rates when compared to NHs. Future studies may include facility or resident characteristics in the study design.

